# Evaluating the relationship between acidogenicity and acid tolerance for oral streptococci from children with or without a history of caries

**DOI:** 10.1080/20002297.2019.1688449

**Published:** 2019-11-07

**Authors:** Jeffrey A. Banas, Erika Takanami, Ryan M. Hemsley, Alissa Villhauer, Min Zhu, Fang Qian, Amber Marolf, David R. Drake

**Affiliations:** a The Iowa Institute for Oral Health Research, University of Iowa College of Dentistry, Iowa City, Iowa, USA; b The Division of Biostatistics and Computational Biology, University of Iowa College of Dentistry, Iowa City, Iowa, USA

**Keywords:** Oral streptococci, acidogenicity, acid tolerance, dental caries

## Abstract

**Background**: Dental caries etiology is attributed to a dysbiotic imbalance within the plaque microbiome leading to a dominance of strong acidogens. Some studies that investigate the link between acidogens and caries quantify the recovery of acid tolerant strains on acid agar as a measure of acidogenic potential. This methodology assumes that acidogenic potential and acid tolerance are directly related.

**Aim**: The validity of that assumption was investigated by statistically evaluating that relationship using streptococci recovered from children with or without a history of dental caries.

**Methods**: Thirty streptococcal isolates were isolated from each of 13 subjects. Acidogenicity was quantified by measuring the terminal pH after overnight growth in Brain Heart Infusion (BHI) and Chemically Defined Medium (CDM). Acid tolerance was quantified by measuring the lowest pH acid agar displaying growth. **Results**: A significant difference in acidogenicity in CDM between levels of acid tolerance was found, but no significant difference in acidogenicity in BHI was noted. Moreover, there were no significant interactions between acid tolerance and caries history on acidogenicity measures in either medium.

**Conclusion**: An ability to grow on acid agar below pH 5.0 is best aligned with strong acidogenicity and best able to distinguish between subjects with differing caries histories.

Understanding microbial contributions to the etiology of dental caries is still an active area of investigation. Stephan and Miller [1] were among the first to document differences in the acidogenic potential of plaque bacteria recovered from carious sites compared to plaque bacteria recovered from sites of sound enamel. Recognition of these differences forms the core of the Ecological Plaque Hypothesis [2,3] which attributes caries development to dysbiosis, leading to dominance by strong acidogens. The degree to which there exists species specificity within this imbalance is still a matter of debate, especially as it applies to the contributions of the mutans streptococci (MS) [4]. Studies that examine the microbiology of dental caries and include quantification of MS typically find that the proportions of MS positively correlate with caries experience but are an imperfect predictor of future caries [5–9]. While this is not surprising given the multifactorial etiology of dental caries, it follows from the Ecological Plaque Hypothesis that recovery of all acid-loving strains, typically done on solid media adjusted to a pH in the range of 5.0 to 5.5, would outperform MS counts in being positively correlated with caries and being a better predictor of future caries risk. However, the reverse is sometimes observed – MS proportions are better correlated with caries history than are the proportions recovered on low pH solid media [10,11]. To better understand these observations, we analyzed the statistical linkage between growth on low pH acid agar and acidogenic potential using streptococcal strains recovered from children with and without a history of dental caries.

## Materials and methods

### Subject recruitment and plaque collection

This pilot project was approved by the Institutional Review Board of the University of Iowa. Subjects were recruited from among patients at the University of Iowa College of Dentistry and Dental Clinics. Inclusion criteria were subjects that ranged from 3 to 10 years with overall good systemic health. Children were designated as positive for caries if they had any active decay or a past history of caries (dmft/DMFT > 0), regardless of severity. Children were designated as negative for caries if they were caries-free and had no history of previous decay. One subject was excluded from the study due to a recent course of antibiotics. Coronal plaque was collected by swabbing exposed coronal surfaces from the full dentition with a sterile, cotton-tipped applicator. The swab with the collected plaque was placed into 1 ml of sterile phosphate-buffered saline (PBS) and transported to the microbiology laboratory.

### Isolation of streptococcal strains

Plaque samples within PBS were mixed by vortexing for 15 seconds at full speed and then sonicated for 20 seconds at a medium setting (10 on the Sonic Dismembrator 60; Fisher Scientific, Waltham, MA). Dilutions were made in PBS and plated on Selective Streptococci Agar (SSA; Hardy Diagnostics, Santa Maria, CA). Plates were incubated in a CO_2_ incubator for 48 hours. Thirty colonies, representing all colonies in a given sector of a plate with well-isolated colonies, were selected and subcultured for isolation on TSA with 5% sheep’s blood (Fisher Scientific; Waltham, MA) to ensure purity. All isolates from all subjects were analyzed for acidogenicity and acid tolerance. Isolates were not, however, analyzed for strain relatedness nor for species designation. The one exception was that isolates were designated as *Streptococcus mutans* if they grew on media selective for the mutans streptococci and displayed a colony morphology consistent with *S. mutans*.

### Measurement of acidogenicity

Relative acidogenicity was determined by measuring the terminal pH after overnight growth in 5 ml of Brain Heart Infusion (BHI) broth and in 5 ml of Chemically Defined Medium (CDM; SAFC Biosciences, Lenexa, KS) plus 1% fetal bovine serum (FBS). Glucose is the carbon source in CDM. The addition of 1% FBS helped improve growth yields that ensure the accuracy of the terminal pH measurements. Measurements were not made for strains that did not fully grow. Streptococcal strains capable of raising the pH from metabolism of alternative carbon sources (i.e. arginine) following the depletion of carbohydrate were evident and accounted for in the pH measurements in BHI. *S. mutans* ATCC 25,175 and *Streptococcus sanguinis* ATCC 10,556 were used as controls to ensure the consistency of pH measurements throughout testing of each group of isolates.

### Measurement of acid tolerance

Relative acid tolerance was determined by plating each streptococcal isolate on a series of BHI agar plates adjusted to a pH of 7.0, 6.0, 5.5, 5.2, 5.0, or 4.8. The pH 4.8 plates solidified but were not of sufficient consistency to allow incubation in an inverted position. The lowest pH at which growth was evident was recorded for correlation with terminal pH measurements of acidogenicity.

### Statistical analyses

In order to maintain independence between study samples and account for variance between subjects in the distribution of isolates that grew at the various pH levels tested, data were combined into three acid tolerance levels: the low-range included isolates that grew at pH 5.0 or lower; the mid-range included isolates that grew at pH 5.2; and the high-range included isolates that grew at pH 5.5 or higher. The terminal pH values (acidogenicity) were averaged for all isolates from a given subject at each of the acid tolerance levels. Within each acid tolerance level, a two-sample t-test was used to examine the difference in acidogenicity in BHI or CDM between subjects with or without caries, while a one-way ANOVA with repeated measures was used to assess the difference in acidogenicity in BHI or CDM among three acid tolerance levels within each caries status group. Additionally, a two-way ANOVA with repeated measures was used to detect an interaction between acid tolerance level and caries status on acidogenicity measures. Statistical analyses were performed using the statistical package SAS® System version 9.4 (SAS Institute Inc., Cary, NC). A significance level of 0.05 was utilized for all tests.

## Results

Fourteen children, seven with a history of caries and seven with a caries-free history, were recruited for this study resulting in the collection of 390 clinical isolates of streptococci. Plaque streptococci could not be isolated from one caries-free subject, due to a recent course of antibiotics, thereby reducing the number of caries-free subjects to six. Table 1 provides details regarding the numbers of subjects, caries status, and numbers of isolates that fully grew in BHI or CDM for inclusion in the statistical analyses. Of the 390 isolates subcultured from plaque samples, the terminal pH in BHI and CDM were determined for 347 (89%) and 264 (68%) isolates, respectively.

**Table 1.** Streptococcal isolates that fully grew in BHI and CDM

	History of caries (n = 4 M; n = 3 F)		Caries-free history (n = 3 M; n = 3 F)		Subtotal by sex	
Total subjects (n = 13)	Grew in BHI	Grew in CDM	Grew in BHI	Grew in CDM	Grew in BHI	Grew in CDM
Male (n = 7)	104	61	80	58	184	119
Female (n = 6)	79	72	84	73	163	145
Subtotal by caries history	183	133	164	131		
Total for each medium					347	264

**Figure 1.**
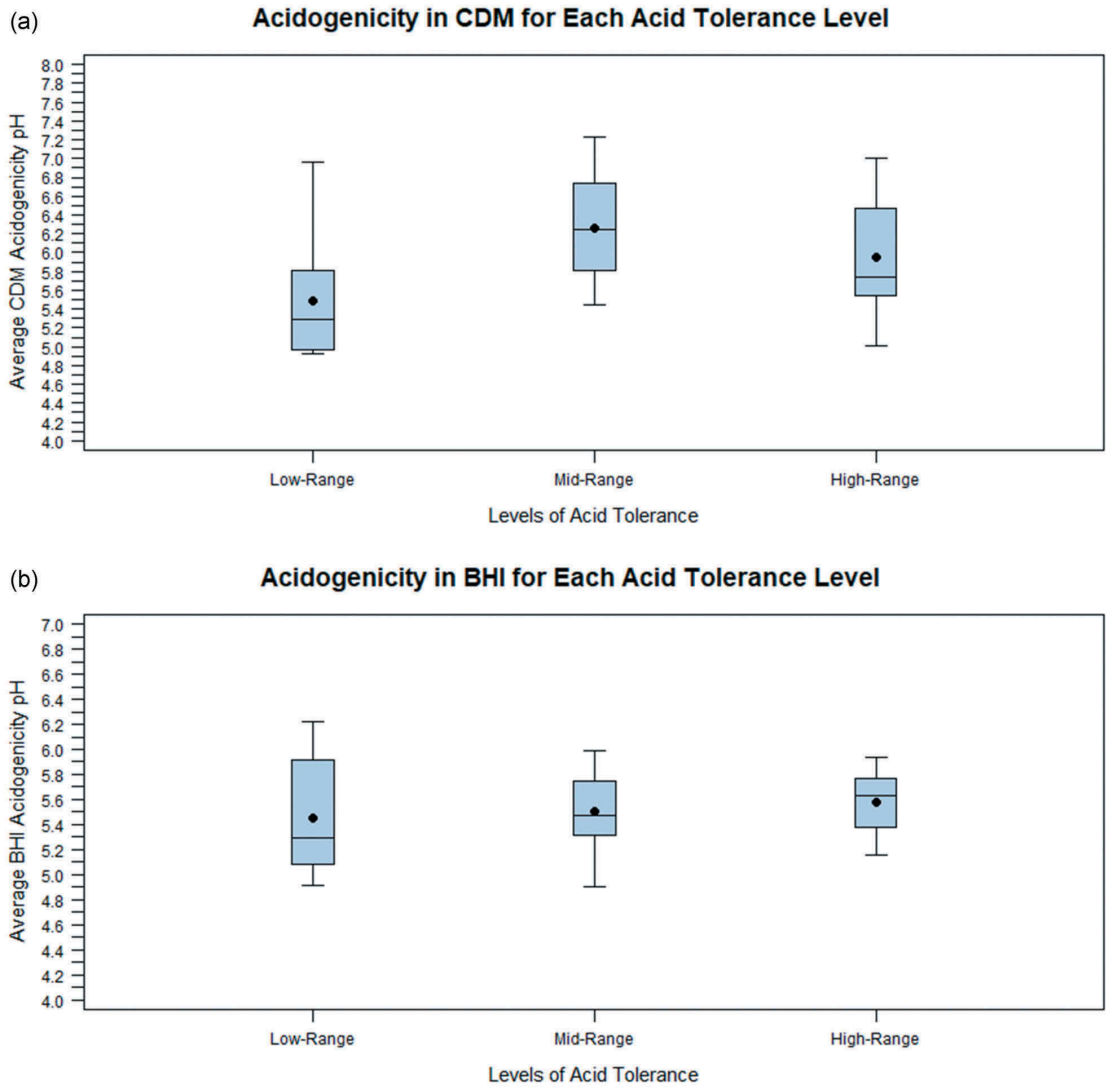
Box plots depicting the difference in acidogenicity between acid tolerance levels for each of the streptococcal strains isolated from children 3 to 10 years of age. The boxes enclose the range from the lower quartile to the upper quartile. The black dot represents the average and the horizontal line represents the median. (a) The relationship between acid tolerance and acidogenicity when terminal pH was measured in CDM, and (b) The relationship between acid tolerance and acidogenicity when terminal pH was measured in BHI

The main objective of the data analysis was to determine if the acid tolerance level impacted acidogenicity measures for the full collection of streptococcal isolates without regard to the caries status of the subjects from whom they were isolated. The data revealed that there was a statistically significant effect of the level of acid tolerance on average acidogenicity in CDM (p = 0.022; Figure 1(a) and Table 2) but not significantly on average acidogenicity in BHI (p = 0.540; Figure 1(b) and Table 2). However, the effect in CDM was significant only when comparing the mid-range (5.2) and low-range (5.0 and lower) acid tolerance levels. No statistically significant difference was found between the high-range (5.5 and higher) acid tolerance level and either the mid- or low-range levels. In fact, the average acidogenicity in CDM at the high-range acid tolerance level was lower, not higher, than that for the mid-range acid tolerance level (pH 5.91 versus 6.26). The acidogenicity in BHI was nearly identical at each acid tolerance level (pH 5.46 for the low-range; 5.50 for the mid-range; and 5.61 for the high-range).

**Table 2.** Average acidogenicity in BHI and CDM for acid tolerancel and caries status

Variable	Mean (SD)	Minimum	Maximum	Median
For average acidogenicity in BHI pH				
Acid tolerance level ≤5.0	5.46 (0.48)^A^	4.92	6.22	5.29
Acid tolerance level 5.2	5.50 (0.32)^A^	4.90	5.99	5.47
Acid tolerance level ≥5.5	5.57 (0.26)^A^	5.15	5.94	5.63
For average acidogenicity in CDM pH				
Acid tolerance level ≤5.0	5.48 (0.62)^A^	4.93	6.96	5.29
Acid tolerance level 5.2	6.26 (0.55)^B^	5.45	7.23	6.24
Acid tolerance level ≥5.5	5.95 (0.63)^A,B^	5.02	7.01	5.73
For average acidogenicity in BHI pH				
Subjects with caries	5.41 (0.33)^A^	4.92	6.15	5.38
Subjects without caries	5.65 (0.35)^B^	4.90	6.22	5.74
For average acidogenicity in CDM pH				
Subjects with caries	5.90 (0.74)^A^	4.93	7.23	5.82
Subjects without caries	5.91 (0.58)^A^	5.00	7.01	5.77

*For each variable, means that do not share a letter are significantly different (p < 0.05)

**Figure 2.**
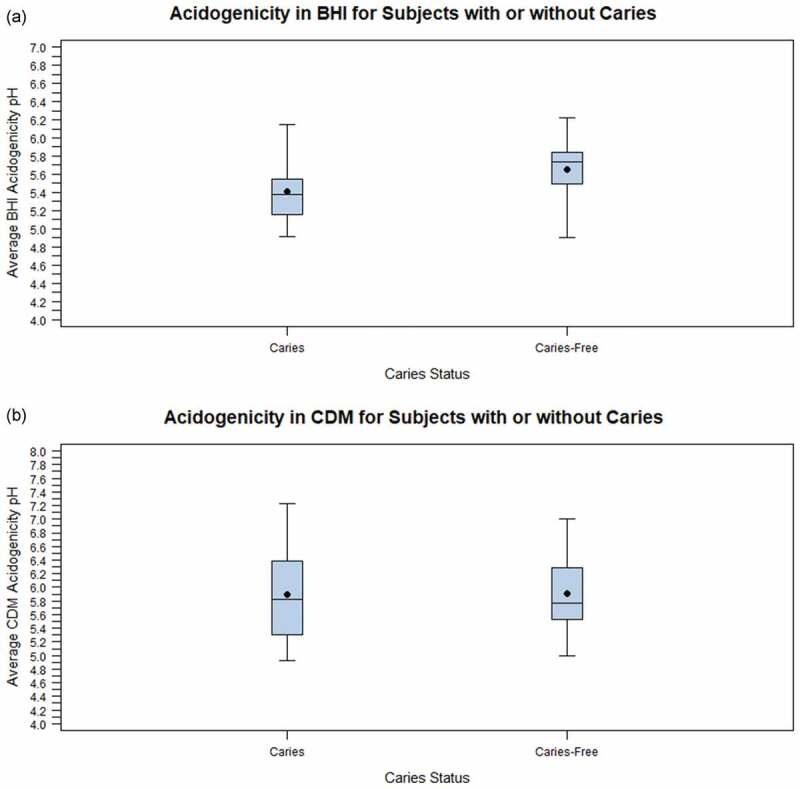
Box plots depicting the difference in acidogenicity for each caries status. The boxes enclose the range from the lower quartile to the upper quartile. The black dot represents the average and the horizontal line represents the median. (a) The relationship between acidogenicity measured in BHI and caries status, and (b) The relationship between acidogenicity measured in CDM and caries status

When the data were analyzed according to each subject’s caries status, there were no statistical differences in the average acidogenicity in CDM (Table 2) or BHI (Table 3) at each of the acid tolerance levels for subjects with or without a history of caries. Nor were there statistical differences in average acidogenicity between subjects with differing caries history for any given acid tolerance level (Tables 3 and 4). However, overall, the average acidogenicity measured in BHI was significantly greater (resulting in a lower average pH) in subjects with caries compared to subjects without caries (5.41 versus 5.65; Figure 2(a) and Table 2). This difference in average acidogenicity was not apparent when measured in CDM (5.90 versus 5.91; Figure 2(b) and Table 2). No significant interaction of caries status with acid tolerance level on average acidogenicity in BHI or CDM was found (p > 0.05 in each instance).

**Table 3.** Comparison of average acidogenicity in BHI for each acid tolerance level and caries status

	Mean average acidogenicity BHI pH (SD)	
Levels of acid tolerance	Subjects with caries	Subjects without caries
Level ≤ 5.0	5.26 (0.42)^A,1^	5.74 (0.44)^A,1^
Level 5.2	5.44 (0.25)^A,1^	5.58 (0.41)^A,1^
Level ≥ 5.5	5.53 (0.28)^A,1^	5.63 (0.25)^A,1^

* For subjects with or without caries, column means with the same letter are not statistically significantly different using the post-hoc Tukey-Kramer test (p > 0.05).

**For each acid tolerance level, row means with the same number are not statistically significantly different using a two-sample t-test (p > 0.05).

**Table 4.** Comparison of average acidogenicity in CDM for each acid tolerance level and caries status

	Mean average acidogenicity CDM pH (SD)	
Levels of acid tolerance	Subjects with caries	Subjects without caries
Level ≤ 5.0	5.49 (0.76)^A,1^	5.47 (0.43)^A,1^
Level 5.2	6.32 (0.60)^A,1^	6.18 (0.52)^A,1^
Level ≥ 5.5	5.88 (0.71)^A,1^	6.04 (0.59)^A,1^

*For subjects with or without caries, column means with the same letter are not statistically significantly different using the post-hoc Tukey-Kramer test (p > 0.05).

**For each acid tolerance level, row means with the same number are not statistically significantly different using a two-sample t-test (p > 0.05).

The percent of isolates from caries-positive and caries-free subjects that grew on acid agar of increasingly low pH were quantified and tabulated in Table 5. As evident from the data in the Table 5, the lower the pH, the better discrimination there is between caries and caries-free subjects. At its most extreme, pH 4.8, the differences are largely, but not exclusively, due to the representation of *S. mutans* (36 of 50 pH 4.8 isolates).

**Table 5.** Percentage and frequency of growth on low pH solid media

		Grew at pH ≤ 5.2		Grew at pH ≤ 5.0		Grew at pH ≤ 4.8	
	Total No. Isolates	No. Isolates [%]	No. Subjects Positive [%]	No. Isolates [%]	No. Subjects Positive [%]	No. Isolates [%]	No. Subjects Positive [%]
Caries subjects (n = 7)	183	126 [68.9]	7 [100]	61 [33.3]	7 [100]	39 [21.3]	6 [85.7]
Caries-free subjects (n = 6)	164	82 [50.0]	5 [83.3]	20 [12.2]	5 [83.3]	11 [6.7]	2 [33.3]

## Discussion

This study was undertaken in order to investigate the possible basis for observations that the average acidogenicity of bacteria from dental plaque samples, as measured by terminal pH in broth media, is more likely to be associated with caries status than is the quantity of oral organisms recovered on acid agar. In a general sense, the ability to produce acid from the metabolism of carbohydrates is linked to an ability to survive within an acidic environment. However, the acidic environment in dental caries is extreme. We speculate that a cariogenic environment requires a collective increase in strong acidogens among plaque bacteria whereas a comparable increase in acid tolerance may be obscured by a broad range of diversity in relative acid tolerance among moderately acidogenic plaque strains.

The data collected in this study affirmed that the average acidogenicity, measured in BHI, of isolates from subjects with caries was significantly greater among isolates from subjects with a history of caries than among isolates from caries-free subjects. This was not true of acidogenicity measurements made in CDM. It is not clear why the difference did not show up in CDM. The range of pH values and standard deviation were much higher for the CDM data but even the median values failed to distinguish subjects with differing caries histories. When the average acidogenicity was calculated for each level of acid tolerance, the trends largely matched expectations – for data from both media – in that the lowest pH (highest acidogenicity) averages were found among isolates that had the highest acid tolerance (grew on acid agar in the low pH range of pH 5.0 or 4.8). These differences were even more pronounced when expressed as median values. However, there was a lack of statistical significance with the exception of the spike in average acidogenicity, measured in CDM, for the mid-range acid tolerance category.

There are multiple methods for assessing acidogenicity and acid-tolerance. It is possible that a stronger correlation between these two phenotypes would be evident if different evaluative protocols had been chosen. However, the choice of terminal pH as a measure of acidogenicity was based on a classical means for defining low pH streptococci [12–14]. Acid tolerance was assessed by growth on acid agar since this method has been used as a convenient means for characterizing and quantifying the acid tolerant component of oral samples. We previously determined that pre-incubation of oral samples in mildly acidic buffer, intended to induce an acid tolerance response, did not elevate the total bacterial recovery on acid agar (data not shown). The rate of acid production is also an important component of acidogenicity. Cariogenic species such as the mutans streptococci are among the most rapid acid producers among oral streptococci and yield a low terminal pH *in vitro* [12–15]. Whether the extent to which total acid production and the rate at which it is produced coincide in other oral species is unknown.

It is possible that a higher-powered study with a larger subject population would find a more consistent statistical relationship between acidogenicity and acid tolerance. But evaluations for cariogenic potential are done on an individual basis. If a statistical relationship is only evident with a large study population, it is unlikely to be of relevance for individual screening. In addition, other data offer a reason to be cautious about the utility of relying on acid tolerance when evaluating the cariogenic potential of dental plaque. When the proportions of isolates capable of growth on the acid agar of lowest pH (5.2, 5.0, and 4.8) were examined, only growth at pH 4.8 provided a clear distinction between subjects with differing caries histories. This suggests that using acid agar of pH 5.2, or even 5.0, have very limited efficacy for this purpose.

In summary, this study was undertaken to investigate the linkage between acid tolerance and acidogenicity. Within the limits of the methods employed, this relationship was found to be inconsistent and media dependent. Studies that employ growth on acid agar as a measure of acidogenic challenge may be best accomplished by using acid agar at a pH below 5.0.
